# Prevalence Rate and Risk Factors of Sexual Assault Among University Students in the Netherlands

**DOI:** 10.1177/08862605231192849

**Published:** 2023-08-23

**Authors:** Alice K. Wellum, Reine M. D. Ramaekers, Jan Schepers, Jos V. M. Welie, Gesa Lange, Petra M. Hurks

**Affiliations:** 1 Maastricht University, The Netherlands

**Keywords:** rape, sexual assault, regression analysis, college students

## Abstract

This research documents the prevalence rate and demographic risk factors for sexual assault among undergraduate and graduate students enrolled at a Dutch university. The present study used a sample of *N* *=* 2,887 students who filled in responses to a campus climate survey about students’ experiences with sexual assault and diverse demographic variables. Results showed that approximately one in four students (25.3%) experience non-consensual sexual touching, and almost one in ten are raped (9.2%). Next, to examine the effects of demographic factors and their interactions on sexual assault, the dataset was divided randomly into two subsamples. Exploratory multiple regression analyses were conducted on the first subsample and confirmatory multiple regression analyses on the second. Variables that increased odds for unwanted sexual touching, rape, and any type of sexual assault were gender; being a member of a student or a study association; having a disability; and being in a relationship (in this context, “any type of sexual assault” refers to any incident that included unwanted touching, attempted rape, or rape). LGBQ+ sexual orientation was significant for any kind of sexual assault and for rape; and being a member of a sport association was significant for any kind of sexual assault and for sexual touching.

## Introduction

A large body of research shows that students in higher education are at risk of sexual assault. Recent estimates suggest that 20% to 25% of students experience sexual assault while enrolled at Western universities (Cantor et al., 2019; Krebs et al., 2011; Muehlenhard et al., 2017; Senn et al., 2014; Wellum et al., 2021). Sexual assault is defined as sexual touching, or attempted or completed penetrative sexual acts, which can be obtained through different methods, including physical force or threat of physical force, incapacitation, verbal coercion, or without active, ongoing, and voluntary consent.

The above-mentioned reports on the number of students who experience sexual assault are alarming, even more so since experiencing sexual assault can lead to a multitude of health consequences, including contracting sexually transmitted disease(s), anxiety, depression, and/or post-traumatic stress disorder (Carey et al., 2018; Kilpatrick et al., 2007; Koss et al., 1994). Besides physical and mental consequences, experiencing sexual assault can also have a negative impact on students’ academic performance. Recent studies among undergraduate students found associations between such experiences and failures and/or delays on assignments, courses, and exams (Stermac et al., 2020), decreased academic attainment, such as lower grade point average (Jordan et al., 2014; Potter et al., 2018; Stermac et al., 2020); and more students who consider dropping out of or indeed drop out of classes (Krebs et al., 2007) or their university program (Baker et al., 2016; Mengo & Black, 2016).

### Sexual Assault at Universities in Europe

Most of the above-mentioned studies were conducted in North American universities, potentially limiting our understanding of sexual assault in other countries. For example, a systematic literature review on sexual assault among 12- to 25-year-olds from 27 European countries found prevalence rates of sexual assault ranging between 2% and 83% (Krahé et al., 2014). This variation in prevalence rates may be attributable to cultural differences, such as the acceptance of physical violence against a dating partner, or the rate of the country’s gender equality with regard to political or economic power (Krahé et al., 2014). As such, Bonar et al. (2022) indicated that it is important to conduct country-specific prevalence rate studies when studying the prevalence of sexual assault among university-age students. The current study, therefore, examines the prevalence and risk factors of sexual assault experienced by university students in the Netherlands. While studies concerning the prevalence of sexual assault among young people in the Netherlands exist (de Haas et al., 2012) only a recent report by Amnesty International describes the prevalence rate of sexual assault among students enrolled in Dutch universities and universities of applied sciences (Driessen & Polet, 2021). The latter study found that the incidences of both unwanted sexual touching and rape were similar to previous studies reported in the United States: for example, the Amnesty International study found that 21% of female participants reported experiencing at least one incident of unwanted sexual touching while enrolled as a student, which is comparable to the study by Flack et al. (2008) who found that 29.8% of female students experienced unwanted sexual touching.

It is reasonable to presume that the similarity in prevalence rates may be attributable to common risk factors found both in the United States and the Netherlands. However, since no study in the Netherlands has examined risk factors associated with sexual assault among the student population, a further aim of this study is to examine a range of common risk factors in relation to sexual assault among the student population at a Dutch university.

### Risk Factors

The dearth of Dutch studies on risk factors associated with sexual assault among university students is highly unfortunate. For while sexual assault will most likely never be eradicated, an important step toward sexual assault reduction, besides reporting prevalence rates, is to identify which risk factors increase the likelihood for students to experience sexual assault (Coulter et al., 2017). An understanding of those risk factors can allow for the implementation of targeted policies, such as the creation or fine-tuning of prevention and support plans. For example, sexual assault prevention training could be targeted specifically at groups who have been shown to be more at risk of experiencing sexual assault.

Previous studies have identified several demographic factors, which may increase the likelihood for students to experience sexual assault. For instance, cis female students have been shown to be consistently more at risk for sexual assault than cis male students (Fisher et al., 2000; Krebs et al., 2011). Sexual orientation also appears to be a risk factor, with gay/lesbian and bisexual students being more at risk for sexual assault than their heterosexual counterparts (Coulter et al., 2017; Martin et al., 2011). However, some studies suggest that gender modifies the effects of sexual identity on sexual assault. For example, Coulter et al. (2017) found that being bisexual increased the likelihood of sexual assault for cisgender men more than cisgender women.

Factors relating to students’ social life have also been cited as risk factors, including participating in “Greek” life (referred to as “fraternities/sororities” in the United States and Canada and “student organizations” in the Netherlands; Franklin, 2016; Humphrey & Kahn, 2000; Minow & Einolf, 2009). Studies on Greek life in the United States have either been conducted on fraternities or sororities, which are traditionally single-sex organizations, with fraternities consisting exclusively of men and sororities consisting exclusively of women. Studies in the United States on sororities have shown that sorority students are more at risk of sexual assault than non-members (Franklin, 2016), even after controlling for alcohol consumption and attendance at events where alcohol was served (Minow & Einolf, 2009). We have included membership in a student association in our study. However, we note that most student associations in the Netherlands have both male and female students.

Being in an intimate relationship has also been cited as a risk factor for experiencing sexual assault (Bhochhibhoya et al., 2021; Cantor et al., 2019; Flack et al., 2007). Studies furthermore suggest that international students may be more at risk to be sexually assaulted than “native” students (Brubaker et al., 2017), potentially due to cultural differences in their home/host university and differences in alcohol use as well as hooking-up culture. However, there are few studies on international students’ experiences outside of the U.S. context. The aforementioned studies point to general tendencies, but it should be noted that results are not always consistent across other studies.

In this study, we investigate whether we can predict which demographic factors increase the likelihood for a student to experience sexual assault while enrolled at a university in the Netherlands. It is also our hope that this study broadens the understanding of sexual assault by examining not only the main effects but also the interaction effects among demographic factors. An interaction effect is “the simultaneous effect of two or more independent variables on at least one dependent variable in which their joint effect is significantly greater (or significantly less) than the sum of the parts” (Lavrakas, 2008, p. 339). Including interaction effects allows for a better understanding of the complex relationship between the predictor factors mentioned above (e.g., gender and year of affiliation) and sexual assault. A previous study that explored interaction effects showed that gender can moderate the effects of race/ethnicity and sexual identity on sexual assault (Coulter et al., 2017). For example, while cisgender women had a higher likelihood of experiencing sexual assault than cisgender men, being bisexual or Black increased the likelihood for a person to experience sexual assault more for cisgender men than for cisgender women. Race or sexual orientation can therefore function as a risk-increasing factor for the gender effect. Another example of the relevance of including interaction effects is a study by Muldoon et al. (2019), who demonstrated that while women are more likely to experience sexual assault than men, this difference appears to decrease with age. Thus, younger age may be seen as a protective factor for the gender effect.

These examples illustrate how overlooking interaction effects may lead to a misrepresentation of the association between predictors and experiencing sexual assault (Lavrakas, 2008). Interaction effects thus have the potential to increase our understanding as to which populations and which circumstances are more likely to experience sexual assault. While we have not included all interaction effects explored in other publications in the current study, we concur with McCauley et al. (2020) that a more comprehensive and nuanced understanding is gained when we include potential demographic factors into one model that includes interaction effects.

## Current Study

This exploratory study analyses a variety of demographic factors within student populations to consider the joint effect of risk factors on three different types of sexual assault: experiencing any kind of sexual assault (which includes sexual touching or attempted or completed sexual penetrative acts), sexual touching, and completed sexual penetration (rape). Since few respondents reported attempts of sexual assault (*N* = 61), attempted sexual assault was only included in the *any sexual assault* variable. Because we did not have an a priori hypothesis about interaction effects, this study is exploratory in nature.

Our research questions are:

(1) What are the prevalence and risk factors of sexual assault in a higher educational institution in the Netherlands?
(2) Does studying interaction effects (next to main effects) increase our understanding of whether demographic factors affect the likelihood of students in a higher educational institution in the Netherlands to experience sexual assault?

## Method

### Participants

A total of *N* = 2,887 students from one Dutch university (Maastricht University or Universiteit Maastricht [UM]) responded to a call for participating in a university-wide “sexual well-being survey.” The call was issued to all students enrolled at Maastricht University in June 2020 (*n* = 17,515) via a pop-up on the students’ laptop or computer screen when they logged into the university environment. Participants who were under the age of 18 years were not allowed to continue with the survey. In total, 409 participants were excluded because they were aged less than 18 years, skipped the question about their age, or did not answer any question in the sexual assault section. The final sample consisted of *n* = 2,478 students, which is 14.15% of the total number of students enrolled at UM in June 2020 (*n* = 17,515). In total, 1,732 female-identifying students (70%) and 708 male-identifying students (29%) were included in our sample. This is roughly proportional to the university demographics, where female students made up about 60% of the population (*n* = 10,527) and male students made up about 40% (*n* = 6,988) of the population of students enrolled in June 2020. At UM, Dutch students made up about 46% of the student population (*n* = 8,050), whereas international students made up around 54% (*n* = 9,465). In our study, 32.4% (*n* = 803) were Dutch students and international students made up 61.5% (*n* = 1,613). Dutch students may be slightly underrepresented in our study because the questionnaire was offered in English only, and not in Dutch. While UM is generally an international university whose language of instruction is English, there are some courses and programs that are offered in Dutch only. Since the invitation to participate in the study was written in English, it is possible that students who were used to communicating in Dutch about their studies ignored the pop-up. The sample of students who reportedly see themselves as nonbinary, genderqueer, or questioning was not included in the analyses as it was too small (*n* = 33). Table 1 shows an overview of demographics.

**Table 1.** Demographics of Random Sample of Respondents of Students at Maastricht University.

Demographic characteristics	*n*	%
Total	2,478	100
Gender		
Female (cis/trans)	1,732	69.9
Male (cis/trans)	708	28.6
Genderqueer students^a^	33	1.3
Decline to state^a^	38	1.5
Sexual orientation		
Heterosexual	1,929	77.8
LGBQ (non-heterosexual)	537	21.8
Decline to state^a^	12	0.5
Member of student association^b^	739	29.8
No member	1,732	69.9
Decline to state^a^	7	0.3
Member of study association^b^	546	22.0
No member	1,925	77.7
Decline to state	7	0.3
Member of sports association^b^	1,077	43.5
No member	1,395	56.3
Decline to state	7	0.3
Disability		
Any disability total	470	19.0
No disability	2,008	81.0
Decline to state^a^	25	1.0
Relationship status		
Serious relationship or ongoing sexual contact	1,880	75.9
Not in a relationship	575	23.2
Decline to state^a^	23	0.9
Nationality		
Dutch	803	32.4
Non-Dutch total	1,613	65.1
Decline to state^a^	2	0.1
Year of affiliation		
Undergraduate	1,748	70.5
Graduate	600	24.2
Other (alumni, exchange students)^a^	104	4.2
Decline to state	18	0.7

a Not included in the analysis.

b Multiple options could be selected for questions on affiliation to student, study, or sport association. Therefore, the numbers do not add up to 100%.

### Measures

An adapted, digital version of the Association of American Universities (AAU) Campus Climate Survey was distributed among the student population of Maastricht University in June 2020. The campus climate survey, issued by the AAU, is the largest of campus climate surveys conducted in the United States, with more than 150,000 students from 27 universities participating. To encourage the response rate in our study, the questionnaire was shortened, with sections on stalking and interpersonal partner violence omitted. Furthermore, in contrast to the original version of the survey, students were not asked about their race (since researchers were not allowed to collect these data in accordance with Dutch law). However, to account for our international student population, students were asked about their nationality. Additionally, for almost all questions students were given the option to “decline to state,” which was a visual way of reassuring students that they did not have to answer sensitive questions. For this study, only the section on general demographic data and sexual assault was included. The average time needed to complete the survey was 90 min.

### Procedure

The study was approved by the Ethics Review Committee of Psychology and Neuroscience of Maastricht University. Students were made aware of the survey in the following ways:

- Students were invited to participate in the survey when logging in to the university’s electronic learning environment for the duration of June 2020.
- Staff members of the various university student resource centers and the university’s diversity and inclusivity department encouraged students to participate via a newsletter and social media channels.
- Members of the university’s student, study, and athletic associations encouraged students to participate in this study via their social media channels.
- Master students in psychology announced the launch of the survey in their classes’ respective Facebook groups.

As an incentive, participants were invited to submit their names to a lottery to win 50 euros. In total, 400 euros was made available in this way. Lotteries are frequently used in digital surveys and are more effective at improving response rates than other forms of incentives (Couper & Bosnjak, 2010). The invitations included a link to the survey, which was administered using the Qualtrics software (Qualtrics and all other Qualtrics product or service names are registered trademarks or trademarks of Qualtrics, Provo, UT, USA. https://www.qualtrics.com). Upon clicking the link, students first received more information about the survey, including the promise of anonymity. Students agreed to this via a consent form and indicated that they were at least 18 years old. A list of support resources was automatically made available to all participants upon completion of the survey.

### Sexual Assault

Sexual assault was assessed via nine questions that employed behaviorally specific questions. The scale included questions on the type of sexual assault, including four questions on non-consensual sexual touching (without penetration; examples include groping and kissing) and four questions on completed rape (oral, vaginal, or anal penetration via a penis, finger, or object). One question was asked about *attempted* (but not completed) non-consensual sexual penetration. For each type of assault—the non-consensual sexual penetration and the non-consensual sexual touching—up to four methods of perpetration could be selected. These included:

- physical force, which is using physical force or threats of physical force. Force could include someone using their body weight to hold you down, pinning your arms, hitting, or kicking you, or using or threatening to use a weapon against the student;
- verbal threats, such as threatening serious non-physical harm or promising rewards;
- incapacitation, that is, passed out, asleep, or incapacitated due to drugs or alcohol, and
- without an active, ongoing, and voluntary agreement, that is, initiating sexual activity despite refusal, ignoring cues to stop, and going ahead without checking in, etc.

Response options were dichotomous: yes/no.

We used three variables in the regression analysis: any kind of sexual assault (participants who had reported experiencing at least one incident of the following: sexual touching, attempted rape, and completed rape [1 = yes; 0 = no]), sexual touching [1 = yes; 0 = no], and completed rape [1 = yes; 0 = no].

#### Demographic Variables

##### Gender Identity

Respondents were asked to indicate an option that best described their gender identity from the following list: woman, man, trans woman (male-to-female), trans man (female-to-male), nonbinary or genderqueer, questioning, and not listed. Three categories were then formed: female-identifying (comprised of respondents who selected “woman” or “trans woman”), male-identifying (respondents who selected “man” or “trans man”), and genderqueer (respondents who selected “nonbinary or genderqueer,” “questioning,” or “not listed”). In the analysis, gender was coded as 1 = woman and 0 = men.

##### Disability

Respondents were asked if they identify as a student with a mental disability or learning disability, a physical disability, both, or none of them. For the analysis, two groups were formed: disability (respondents who had selected “mental or learning disability,” or “physical disability,” or both; this was coded as 1) or no disability (respondents who had selected “none of the above”; coded as 0).

##### Sexual Orientation

Respondents were asked if they considered themselves to be heterosexual or straight, gay or lesbian, bisexual, asexual, queer, questioning, or not listed. This yielded two groups: heterosexual (students who had indicated “heterosexual or straight”; coded as 0) and LGBQ (students who had selected “gay or lesbian,” “bisexual,” “asexual,” “queer,” “questioning,” or “not listed”; coded as 1).

##### Student, Study, or Sport Associations

The Netherlands has various student associations, which are similar to North American fraternities and sororities. In the case of student associations, membership needs to be applied for and is approved after an initiation period, and members have close social ties, often living together and spending a significant amount of their social lives together, such as by hosting parties. Similar to student associations, study associations can host parties and organize events, thereby ensuring that their members spend a lot of time together. However, membership is not dependent on an initiation period, and fees are lower. Study associations are often affiliated with a faculty or department; they may also host faculty/department-specific events, such as debates and lectures. Sport associations are associations that enable members to come together and play a particular sport, participate in tournaments, and often organize social events. In some cases, membership needs to be applied for though in most this is not the case. Respondents were asked: “Since you have been a student at Maastricht University, have you been a member or participated in any of the following?” and could choose from the following answers (multiple answers were possible): student association, study association, UM Sports or UM student sport association, other extracurricular activities affiliated with Maastricht University, none of the above, and decline to state. The following coding was adhered to in the analysis: member of a (student/study/sport) association: 1 = yes and 0 = no.

##### Romantic/Sexual Relationships

Respondents could indicate if they were in a long-term romantic or sexual relationship since enrolling at the university. Response choices were “steady or serious relationship,” “other ongoing relationship involving physical or sexual contact,” and “none of the above.” Two groups were made based on responses: 1 = in a relationship (“steady or serious relationship” and “other ongoing relationship involving physical or sexual contact”); 0 = not in a relationship (“none of the above”).

##### Age

Respondents were able to select their age from the following categories: 18, 19, 20, 21, 22, 23, 24, 25, 26, 27, 28, 29, and 30+ years. From these, we created two groups: aged 18 to 21 years (1) and 22 years and older (0).

##### Nationality

Few studies have examined to what extent non-national students are more or less likely to experience sexual violence. Due to governmental rules, researchers in the Netherlands are currently not allowed to collect data on race or ethnicity. However, this research took place at an international university where more than half (55%) of students are from a non-Dutch background. Based on answers to open questions, two groups were made: 1 = non-Dutch nationality and 0 = Dutch nationality.

### Data Analysis

Analyses were conducted using the BM SPSS Statistics for Windows, version 27 (IBM Corp., Armonk, N.Y., USA). Descriptive variables for all participants were reviewed first. For our first research question concerning prevalence rates of sexual assault, the entire dataset was used. For our second research question, whether interaction terms affect the outcome of sexual violence, we decided to split the sample randomly into two equally sized samples. We performed one set of analyses on the exploratory data set, and then we tested the resulting model on the confirmatory data set. We decided to use this method because employing one model alone may yield incorrect results due to chance capitalization and fitting random variation. By using a random split, the random variation differs across both samples (the exploratory model and the confirmation model). Therefore, we can assume that the effects found in the exploratory model due to chance capitalization will not be confirmed in the confirmatory model.

For the exploratory analyses, three stepwise logistic regression approaches were used, one for each of the dependent variables of sexual assault outcome (any kind of sexual assault, sexual touching, and rape). For the exploratory regression analysis, all main and two-way interaction effects were included and then the model was stripped down via stepwise deletion by removing predictor variables that were not significant (for the exploratory condition statistical, significance was set at .10). This was done first for all two-way interaction terms and then for the main factors. First-order terms that were involved in a higher-order interaction term were not removed from the regression model.

For the confirmatory analyses, all significant terms from the exploratory model were included, but non-significant interaction terms were removed because their inclusion affects the interpretation of first-order (i.e., main effect) terms. For the confirmatory condition, statistical significance was set at .05.

After the exploratory analysis, the confirmatory analysis was used to check whether our results from the initial analyses could be confirmed.

## Results

### Prevalence of Sexual Assault

Table 2 provides an overview of the prevalence rate of sexual assault at Maastricht University. 26.2% of respondents reported experiencing at least one incident involving any kind of sexual assault (sexual touching, attempted rape, or rape). Female-identifying students (32.9%) reported higher rates than male-identifying students (9.7%). 25.3% of respondents reported experiencing at least one incident involving sexual touching (female-identifying students: 31.5%; male-identifying students: 9.5%). Additionally, 9.2% of respondents reported at least one incident of rape. Female-identifying students were more likely to report experiencing rape than male-identifying students (11.8% vs. 2.4%).

**Table 2.** Prevalence Rate of Experienced Sexual Violence Among University Students at Maastricht University in the Netherlands.

Type of Sexual Assault	Total		Female Students		Male Students	
	*n*	%	*N*	%	*n*	%
Any kind of sexual assault	655	26.2	570	32.9	69	9.7
Sexual touching	628	25.3	545	31.5	67	9.5
Sexual penetration	227	9.2	204	11.8	17	2.4
Total *n*	2,478		1,732		708	

*Note*. For comparability with other studies, we have separated the results by gender.

### Influence of Demographic Factors, and Interactions Among Them, on Sexual Assault

#### Exploratory Analysis

After conducting a stepwise regression on one-half of the dataset (exploratory analysis), the following main and interaction terms were found to be promising predictor variables. We considered promising predictor variables to be those that have *p* < .10. Table 3 gives an overview of main and interaction terms. In line with our initial idea that interactions might be relevant, our exploratory analysis showed several significant interaction terms: for example, disability × study association, disability × nationality, gender × sexual orientation, sport association × study association were highly significant for any kind of sexual assault and sexual touching, but not rape.

**Table 3.** Logistic Regression Models of the Exploratory Analysis for Three Types of Sexual Assault, Including Simple Effects and Interaction Terms.

Variables	Model 1: Any Kind of Sexual Assault			Model 2: Sexual Touching			Model 3: Rape		
	*b*	*SE*	Sign.	*b*	*SE*	Sign.	*b*	*SE*	Sign.
Gender (ref. male)	**1.449**	**0.303**	**<.001**	**1.449**	**0.302**	**<.001**	**1.768**	**0.380**	**<.001**
Disability (ref. no disability)	−0.279	0.360	.439	−0.118	0.359	.742	**2.515**	**0.674**	**<.001**
Sexual orientation (ref. heterosexual)	**2.015**	**0.469**	**<.001**	**1.220**	**0.523**	**.020**	**0.723**	**0.242**	**.003**
Student association membership (ref. non-member)	**0.796**	**0.231**	**<.001**	**0.712**	**0.231**	**.002**	**1.233**	**0.257**	**<.001**
Study association membership (ref. non-member)	0.234	0.358	.513	0.216	0.352	.538	**0.940**	**0.337**	**.005**
Sport association membership (ref. non-member)	0.301	0.364	.408	0.181	0.363	.618			
Relationship (ref. no relationship)	0.210	0.248	.397	0.145	0.245	.556	**1.679**	**0.531**	**.002**
Nationality (ref. Dutch)	−**0.692**	**0.255**	**.007**	−**0.508**	**0.217**	**.019**			
Age (ref. 22+)	−**0.912**	**0.445**	**.041**	−**0.680**	**0.398**	**.088**	0.245	0.247	.322
Age (ref. 22+) × gender (ref. male)	**0.841**	**0.425**	**.048**	**0.795**	**0.429**	**.064**			
Age (ref. 22+) × sexual orientation (ref. heterosexual)	−0.700	0.355	.049						
Age (ref. 22+) × nationality (ref. Dutch)	*b*	0.308	.081						
Age (ref. 22+) × study association (ref. non-member)							−**1.313**	**0.489**	**.007**
Disability (ref. no disability) × study association (ref. non-member)	**1.370**	**0.474**	**.004**	**1.095**	**0.465**	**.018**			
Disability (ref. no disability) × student association (ref. non-member)							−**1.575**	**0.518**	**.002**
Disability (ref. no disability) × nationality (ref. Dutch)	**1.028**	**0.401**	**.010**	**0.842**	**0.402**	**.036**			
Disability (ref. no disability) × relationship (ref. no relationship)							−**1.575**	**0.518**	**.002**
Gender (ref. male) × sexual orientation (ref. heterosexual)	−**1.010**	**0.482**	**.026**	−**1.160**	**0.480**	**.016**			
Nationality (ref. Dutch) × study association (ref. non-member)	**0.693**	**0.378**	**.067**	**0.729**	**0.373**	**.051**			
Relationship (ref. no relationship) × sport association- (ref. non-member)	**0.697**	**0.385**	**.070**	**0.726**	**0.384**	**.058**			
Sport association (ref. non-member) × student association (ref. non-member)	−**0.683**	**0.317**	**.031**	−**0.574**	**0.317**	**.070**			
Sport association (ref. non-member) × study association (ref. non-member)	−**1.101**	**0.352**	**.002**	−**1.044**	**0.350**	**.003**			
Student association (ref. non-member) × study association (ref. non-member)	**0.645**	**0.379**	**.089**	**0.702**	**0.377**	**.063**			
Sport association (ref. non-member) × study association (ref. non-member)	−**1.1.01**	**0.352**	**.002**	−**1.044**	**0.350**	**.003**			

*Note*. Significant effects have been written in bold.

#### Confirmatory Analysis

Table 4 shows three logistic regression models for the three types of sexual assault: any kind of sexual assault, sexual touching, and non-consensual sexual penetration, and any kind of sexual assault. Non-significant interaction effects were eliminated.

**Table 4.** Logistic Regression Models of the Confirmatory Analysis for Three Types of Sexual Assault.

Variables	Model 1: Any Kind of Sexual Assault				Model 2: Sexual Touching				Model 3: Rape			
	*b*	*SE*	Sign.	Exp (*B*)	*b*	*SE*	Sign.	Exp (*B*)	*b*	*SE*	Sign.	Exp (*B*)
Gender (ref. male)	**1.446**	**0.216**	**<.001**	**4.247**	**1.409**	**0.219**	**<.001**	**4.091**	**1.403**	**0.364**	**<.001**	**4.069**
Disability (ref. non-disabled)	**0.558**	**0.176**	**.002**	**1.747**	**0.612**	**0.180**	**<.001**	**1.844**	**0.796**	**0.239**	**<.001**	**2.216**
Sexual orientation (ref. heterosexual)	**0.376**	**0.180**	**.036**	**1.457**	0.300	0.183	.101	1.350	**0.664**	**0.244**	**.007**	**1.943**
Student association membership (ref. non-member)	**0.734**	**0.155**	**<.001**	**2.083**	**0.747**	**0.156**	**<.001**	**2.111**	**0.616**	**0.225**	**.006**	**1.852**
Study association membership (ref. non-member)	**0.634**	**0.177**	**<.001**	**1.885**	**0.620**	**0.179**	**<.001**	**1.859**	**0.672**	**0.235**	**.004**	**1.957**
Sport association membership (ref. non-member)	**0.342**	**0.149**	**.021**	**1.408**	**0.374**	**0.151**	**.013**	**1.454**				
Relationship (ref. no relationship)	**0.597**	**0.189**	**.003**	**1.814**	**0.541**	**0.195**	**.005**	**1.718**	**1.424**	**0.406**	**<.001**	**4.153**
Nationality (ref. Dutch)	0.132	0.168	.433	1.141	0.157	0.171	.356	1.170				
Age (ref. 22+)	0.207	0.149	.166	1.230	0.223	0.152	.141	1.250	−0.050	0.221	.819	0.951

*Note*. Significant main effects have been written in bold.

### Risk Factors and Interaction Effects

Most main factors that were significant in the exploratory analysis also showed up in the confirmatory analysis. In the confirmatory analysis, the following variables were significant across all three models: gender (any kind of sexual assault: *OR* = 4.25, *p* < .001; sexual touching: *OR* = 4.09, *p* < .001; and rape: *OR* = 4.07, *p* < .001), disability (any kind of sexual assault: *OR* = 1.75, *p* < .002; sexual touching: *OR* = 1.84, *p* < .001; and rape: *OR* = 2.22, *p* < .001), being a member of a student association (any kind of sexual assault: *OR* = 2.08, *p* < .001; sexual touching: *OR* = 2.11, *p* < .001; and rape: *OR* = 1.85, *p* < .006), being a member of a study association (any kind of sexual assault: *OR* = 1.89, *p* < .001; sexual touching: *OR* = 1.86, *p* < .001; and rape: *OR* = 1.96, *p* < .004), and being in a relationship (any kind of sexual assault: *OR* = 1.81, *p* < .003; sexual touching: *OR* = 1.72, *p* < .005; and rape: *OR* = 4.15, *p* < .001). Gender had the highest odds across all models. Sexual orientation was significant for any kind of sexual assault (*OR* = 1.46, *p* < .036) and rape (*OR* = 1.94, *p* = .007); and being a member of a sport association was significant for any kind of sexual assault (*OR* = 1.41, *p* = .021) and sexual touching (*OR* = 1.45, *p* = .013). There were no significant interaction effects in the confirmatory analysis.

## Discussion

### Overall Prevalence Rates

Overall, around one in four students (26.2%) in our study reported experiencing at least one kind of sexual assault; around one in four students (25.3%) reported experiencing non-consensual sexual touching; and one in ten students (9.2%) reported experiencing rape. These numbers are comparable with other studies on sexual assault in higher education conducted in North America (e.g., Muehlenhard et al., 2017; Senn et al., 2014) and a recent study conducted in the Netherlands (Driessen & Polet, 2021). In the latter study, 21% of student respondents indicated that they experienced sexual touching and 11% indicated that they had experienced non-consensual sexual penetration while enrolled at a university or at a university of applied sciences in the Netherlands. However, that study used a smaller sample (*n* = 1,059) than our study and was not peer-reviewed.

### Predictors of Sexual Assault

Identifying the prevalence rates and predictors of sexual violence is seen as an important first step to sexual violence prevention (Coulter et al., 2017). As shown in previous studies in North America, our study showed that female students (Coulter et al., 2017; Johnson et al., 2016) and students who were a member of a student association or a study association (Franklin, 2016; Humphrey & Kahn, 2000; Mellins et al., 2017; Minow & Einolf, 2009), carried a higher risk for experiencing sexual assault. As with previous studies (e.g., Mellins et al., 2017), in the current study, students who were members of student associations were more likely to experience sexual touching than rape. Our finding that students in a relationship at some point during their study were more at risk for rape has also been reported in other studies (Bhochhibhoya et al., 2021).

Our study found that having an LGBQ+ sexual orientation was associated with higher odds for experiencing any kind of sexual assault and rape. An exception is sexual touching, which was significant in the exploratory but not in the confirmatory analysis. A possible reason for this is that the current study did not nuance between different LGBQ+ students. Johnson et al. (2016) found that lesbians are no more likely than heterosexuals to experience any kind of sexual assault, sexual touching, or rape. Therefore, using LGBQ+ as a broad category without further differentiation may obscure the nuances concerning the differential risk group as well as types of sexual assault, which became evident when using a stricter *p*-value in the confirmatory analysis.

Despite indications that non-national students are more at risk than national students for sexual assault (e.g., Bonistall Postel, 2020), in the current sample international students were not found to be at a greater risk for experiencing sexual assault than students from the Netherlands. A reason for this may be that at the university where this study was conducted, the proximity to the German and Belgian border means that a large percentage of non-Dutch students are either Belgian or German and are used to living close to the border. Indeed, Bonistall Postel (2020) suggests that the increased risk of international students to sexual assault may be due to different views on consent, different attitudes to binge drinking (in terms of frequency and types of drink), and different kinds of socialization. It is likely that these reasons do not apply equally to Belgian and German students at Maastricht University, since they are culturally quite similar to Dutch students (and certainly more so than students from countries outside the European Union). Furthermore, research should examine whether non-European international students at Dutch universities are indeed more at risk for sexual assault compared to students from the Netherlands and neighboring countries.

### Interaction Effects

We did not find any significant interaction effects that were consistent across both the exploratory and the confirmatory analyses. Although this may be initially surprising, as other studies with comparable demographic variables reported finding them, a closer look at the studies that examined interaction effects shows that these studies used either interaction effects made up of at least one variable which was not included in the current study, such as alcohol (Benson et al., 2007) or race (Coulter et al., 2017), or nuanced variables by for example differentiating between lesbians, gays, and bisexuals (Coulter et al., 2017), instead of grouping them as LGBQ+ as we did.

### Limitations and Recommendations for Future Research

There are several limitations that need to be taken into account when interpreting these results. One limitation is that this study did not include important risk factors that were previously linked to sexual assault, such as alcohol (Benson et al., 2007), or previous sexual victimization (O’Connor et al., 2021). Especially the non-inclusion of alcohol may have influenced the outcome, as alcohol has been consistently associated with an elevated risk for sexual violence, victimization, and perpetration (Basile et al., 2021; Minow & Einolf, 2009). However, even when controlling for alcohol, studies show that demographic risk factors (such as affiliation with a student association; Minow & Einolf, 2009) still have an effect.

Unfortunately, in this study, only respondents who had experienced sexual assault were given a follow-up question as to whether they or the perpetrator had consumed alcohol. The results showed that alcohol was involved in more than half of the cases of sexual assault, but since students who had not experienced sexual assault did not talk about their alcohol consumption, alcohol could not be included as a risk factor.

A further limitation our study had was the not splitting up trans- and cis-gender students into different sub-categories for the gender variables. Recent research has shown that trans individuals are more at risk for experiencing sexual violence than their cis counterparts (e.g., Cantor et al., 2015; Coulter et al., 2015, 2017; Griner et al., 2020); however, our sample size of trans students was too small to create separate sub-variables for trans students. We strongly recommend that future research on risk factors of sexual assault in the Netherlands should focus on the experience of trans and gender-nonconforming students in particular.

At the time the survey was administered, 46% of students were classified as Dutch students, whereas only 32.4% of respondents who were included in the analysis were classified as Dutch. As we have mentioned previously, the fact that the survey was advertised exclusively in English may have inadvertently excluded students who were enrolled in a mostly Dutch-language study. Furthermore, campus climate surveys taken at Dutch institutions should translate the survey into Dutch to ensure that a more representative sample of Dutch students participates.

Another limitation concerns the missing outcome values. If these are related to unknown factors, it is possible that they may be biased. A final limitation was that to encourage students to complete the survey, the sections on intimate partner assault and stalking were not included. However, these are evidently issues of importance, and more research on their prevalence among Dutch university students is needed.

## Conclusion

This study represents the first study that examined both prevalence rates and risk factors of sexual assault reported by students enrolled at Maastricht University in the Netherlands. Given that a large number of university students are affected by sexual assault during their studies in every country where prevalence studies have been undertaken, it is crucial that we gain a better understanding of specific risk factors. However, while continuing to study this complex problem, it should not be forgotten that vast numbers of students face sexual violence right now. Hence, we strongly suggest that research goes hand-in-hand with comprehensive prevention strategies based on established best practices that can serve to reduce number of sexual violence among the student population in higher education.
